# Deep Ensemble Model for Classification of Novel Coronavirus in Chest X-Ray Images

**DOI:** 10.1155/2021/8890226

**Published:** 2021-01-05

**Authors:** Fareed Ahmad, Amjad Farooq, Muhammad Usman Ghani

**Affiliations:** ^1^Department of Computer Science, University of Engineering and Technology, Lahore 54890, Pakistan; ^2^Quality Operations Laboratory, Institute of Microbiology, University of Veterinary and Animal Sciences, Lahore, Pakistan

## Abstract

The novel coronavirus, SARS-CoV-2, can be deadly to people, causing COVID-19. The ease of its propagation, coupled with its high capacity for illness and death in infected individuals, makes it a hazard to the community. Chest X-rays are one of the most common but most difficult to interpret radiographic examination for early diagnosis of coronavirus-related infections. They carry a considerable amount of anatomical and physiological information, but it is sometimes difficult even for the expert radiologist to derive the related information they contain. Automatic classification using deep learning models can help in better assessing these infections swiftly. Deep CNN models, namely, MobileNet, ResNet50, and InceptionV3, were applied with different variations, including training the model from the start, fine-tuning along with adjusting learned weights of all layers, and fine-tuning with learned weights along with augmentation. Fine-tuning with augmentation produced the best results in pretrained models. Out of these, two best-performing models (MobileNet and InceptionV3) selected for ensemble learning produced accuracy and FScore of 95.18% and 90.34%, and 95.75% and 91.47%, respectively. The proposed hybrid ensemble model generated with the merger of these deep models produced a classification accuracy and FScore of 96.49% and 92.97%. For test dataset, which was separately kept, the model generated accuracy and FScore of 94.19% and 88.64%. Automatic classification using deep ensemble learning can help radiologists in the correct identification of coronavirus-related infections in chest X-rays. Consequently, this swift and computer-aided diagnosis can help in saving precious human lives and minimizing the social and economic impact on society.

## 1. Introduction

Microbes live within us, on us, and all around us in the environment. Some microbes live in harmony with birds and other animal species, but can cause disease in humans, as demonstrated by the number of zoonotic infections that transmit from animals to humans [1]. The scale, scope, and global impact of zoonoses threaten not only the well-being of humans and animals but also worldwide safety and health [2]. Roughly 1500 pathogens are known to cause infections in humans [3], and out of these 61% of the identified and 75% of the evolving contagious diseases in human beings are of zoonotic origin [2, 4]. It is estimated that contagious diseases cause almost 16% of all mortalities and 44% of mortalities in low-income republics [5]. According to USDA, the yearly economic loss due to foodborne illnesses in USA was estimated between $10 billion and $83 billion [6]. Every year zoonotic diseases cause 2.7 million deaths and 2.5 billion illnesses in humans [7]. Emerging zoonotic infections are responsible for many significant and devastating outbreaks [8].

Coronavirus is a zoonotic pathogen [9] that infects the cells of human airways and as a result causes pneumonia and severe respiratory infections, kidney failure, and even death [10]. The pathogen can survive in the air and other surfaces from several hours to several days [11]. Health officials are of the view that the pathogen spreads through sneezed and coughed droplets, but some researchers are of the view that its airborne transmission is also taking place [12–14]. A thrilling research predicts that due to sneeze, the particles of the pathogen can travel up to 27 feet [15]. The ease of its propagation, small infectious dosage, and a high volume for ailment/death make it a potential candidate for biological warfare [16, 17].

In the US and Europe, the disease is widely prevalent, and millions of people are affected, and thousands have died due to the disease [18]. In the US an estimated 2,155,572 cases are reported and 117,632 deaths as of 19 June 2020 [19]. The top US infectious disease experts predict that lethal pathogens might kill up to 2.4 million people in the country [20]. In European countries, such as the UK, Italy, Spain, France, and Germany, it is assessed that around 1.2 million people got infected and 0.1 million of them lost their lives due to the disease [18]. British health officials are of the view that the pathogen could infect 80% of the population, and 0.5 million individuals could die due to the pandemic [21]. All over the world, billions are either living in self-quarantine or under lockdown by the governments. If the pathogen is permitted to proceed its way, healthcare infrastructure will be overwhelmed, economies will collapse, and millions of precious lives will be lost [22, 23].

Coronaviruses are recognized to infect birds and mammals, as well as cats, pigs, dogs, bats, chickens, pangolins, and cattle [24]. Research suggests that the novel coronavirus is transmitted to humans either through bats or pangolins [25]. No direct association among humans and other species is recorded; however, the pathogen is a highly mutated microbe, which can easily cross the species obstacle [26, 27].

The most frequently used methods for identification of novel coronavirus is RT-PCR [28] and ELISA [23]. The essential screening procedure applied for recognizing COVID-19 cases is RT-PCR, which can identify the virus's RNA from lower respiratory tract samples. These samples are obtained in various ways, such as oropharyngeal or nasopharyngeal swabs. Though RT-PCR is considered as a golden standard for the pathogen identification [29, 30], it is really time taking, sensitive, and complex manual method.

An alternative procedure that is also used for novel coronavirus screening is a radiography test, where radiograph images (e.g., CT-Scans or X-ray) are attended and examined by radiologists to observe evident signs connected with COVID-19. The early research studies showed that patients show irregularities in chest radiograph images that are illustrative of those affected with SARS-CoV-2 viral disease [31, 32], with some proposing that radiograph test could be applied as a principal tool for the virus's screening in affected regions [29]. There are numerous benefits of using radiography imaging for coronavirus screening during the pandemic, especially in heavily affected and resource-constrained areas. Firstly, these tests are readily available and accessible in our healthcare infrastructure. Secondly, they can be carried out swiftly for COVID-19 patients, which makes them a real complement to PCR examination (in some instances, even displaying greater sensitivity) [33], especially in areas with high volumes of patients, or even as stand-alone when a viral examination is not a choice due to low supplies. Nevertheless, the most significant bottleneck encountered is the demand for experienced radiologists to evaluate the radiograph images, as the visual indicators can be complicated [34]. However, computer-aided models can help radiologists to quickly and precisely assess radiograph images to identify pathogenic infections that cause COVID-19. There is a vital need to devise computer-aided solutions using easily accessible and available chest X-ray imaging to assist in the battle against the coronavirus pandemic. In recent times, the machine and deep learning techniques can facilitate in quick diagnosis, prevention, and treatment of the infections caused by coronavirus [35–38].

Lately, deep learning-based techniques have provided continuous progress in respect of efficiency and prediction accuracy. These models have shown superior generalization ability to solve complex problems of computer-vision, especially in the biological and medical fields such as medical image identification [39], organs recognition [40], bacterial colony classification [35, 39], and disease identification [41]. CNNs have shown exceptional results in medical imaging domain than other traditional networks [41, 42].

With the emergence of huge-size categorized data for training, ImageNet [43], efficient overfitting preventing technique (“dropout” [44], and convolutional neural networks (e.g., SqueezNet [45], VGGNet [46] AlexNet [47], ShuffleNet [48], GoogleNet [49], Xception [50], ResNet [51], inceptionv3 [52], and NasNet [53]) have revealed excellent result on image classification problems [54]. The foremost benefit of deep CNNs for image classification tasks is that the complete deep architecure is trained throughout, from initial raw pixels to final classes, which reduces the necessity for devising a handcrafted feature extractor. However, the principal shortcomings of the deep CNN model are as follows: (i) a robust GPU machine is needed to expedite the training process; (ii) a huge volume of training examples with labels is needed for learning of the weights.

Sometimes, individuals with meager processing power and huge training datasets, unfortunately, cannot receive the benefit of robust CNN models. The familiar deep learning models, such as AlexNet [47], with 5 convolution layers, 3 fully connected layers, and a SoftMax layer, comprise of almost 60 million parameters. Few deeper models, such as VGGNet, with 16 and 19 layers [46] and GoogleNet with 22 layers [49] can accomplish enhanced performance and possess an even greater number of parameters. Parameter learning from limited training examples will produce overfitting, even if its prevention methods are utilized. So, how to implement CNNs to accomplish alike performance on modest datasets as on massive datasets is a pretty challenging task [55].

A simple solution for applying pretrained deep models on an insignificant amount of dataset is transfer learning [56], which removes the last few layers of a pretrained deep architecture and fine-tune it on a unique dataset. The method can be very effective if proper hyperparameters are set, and efficient fine-tuning approaches are adapted. Another effective approach that is also being used for classification of various image classification tasks is ensemble learning. It is generally a machine learning technique, also used in deep learning in which more reliable predictive performance is attained by merging the features from numerous deep designs into a classifier of good quality.

**Figure 1:**
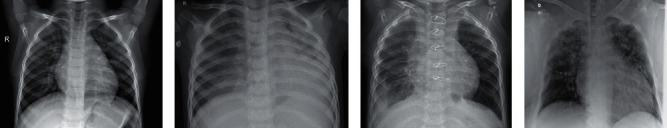
Example posteroanterior chest radiograph images of (a) normal, (b) viral, (c) bacterial, and (d) novel coronavirus infection in COVID dataset.

In the proposed research, we organized a relatively larger and balance image dataset relating to patients with viral, bacterial, and novel coronavirus-related infections in X-rays, along with X-rays of healthy persons (see Figure 1). These images are classified using transfer learning along with fine-tuning on pretrained models to illustrate that these pretrained networks can produce fantastic results when data is limited. Apart from applying transfer learning and fine-tuning, we also used traditional data augmentation techniques such as reflection and rotation [57], which resolves the insufficient training data matter by enhancing the training dataset with transformed original instances. Finally, we suggest a deep ensemble learning model comprising of MobileNet and InceptionV3 models, which generates far more superior results than currently available fine-tuned pretrained models. The devised design attains excellent performance on image dataset relating to novel coronavirus and other related chest infections in X-rays.

We sum up the contributions of the research as follows:

- We have used a balanced and larger dataset with 1050 images in each of the four categories, i.e., normal, viral, bacterial, and COVID-19.
- We propose a deep model for feature fusion of deep models using ensemble learning. The model further integrates transfer learning, fine-tuning, augmentation, and hyperparameter tuning into one design.
- The proposed model further applies 4-fold cross-validation to authenticate the performance of the suggested methodology.
- Test dataset with 50 images in each of the four categories was also kept to see generalizability of the model.

Section 2 puts forward a literature review.  Section 3 presents a complete examination of the proposed methodology, including sections such as an overview of CNN architecture, data augmentation, deep CNN models, and transfer learning.  Section 4 discusses the results achieved after applying various deep models and comparing them with the suggested methodology. Lastly, Section 5 provides the conclusion of the article.

## 2. Related Work

Deep learning and image processing algorithms in biomedical image analysis and processing have produced exceptional results especially in the area of chest radiology. These techniques are frequently applied to conduct pulmonary tuberculosis classification [58] and lung nodule identification [59]. We can say that there are various approaches that are successfully applied for automatic classification of these disease-causing pathogens. There are, among others, different approaches such as CNNs, ensemble learning, and feature extraction. A short review of some important contributions from the existing literature is presented.

Stephen et al. [60] present a deep model trained from the beginning to detect and classify pneumonia in chest X-rays images [61]. The ConvNet model extracts related features from images and utilizes it to classify the disease. The dataset comprises of 64% training and 36% validation data. The model achieves an accuracy of 93.73% with a loss of 0.1835 on a small dataset with the help of fine-tuning, hyperparameter tuning, and augmentation.

In [62], a deep learning model based on Compressed Sensing for computer-aided disease detection on chest X-ray images was suggested to support the doctors. In this study, the dataset used 5863 images of normal or abnormal patients from Kaggle. Extensive simulation results have revealed that the recommended procedure allows the classification of pneumonia (abnormal/normal) with 97.34% foresight accuracy.

Ayan and Ünver [63] propose an early pneumonia diagnosis from chest X-ray images based on VGG16 and Xception pretrained networks. The dataset consisted of 1583 images pertaining to healthy patients and 4273 images relating to pneumonia patients. The results show that Xception generates an accuracy of 87% and VGG16 produces an accuracy of 82%. The confusion matrix shows that each network has its own capabilities, Xception is more successful in detecting pneumonia outcomes, and Vgg16 performs better for normal cases.

Varshni et al. [64] applies pretrained deep learning models, i.e., (ResNet50, Xception, DenseNet121, VGG-16, DenseNet-121, and DenseNet169) for feature extraction, and classification is performed using various machine learning models, i.e., (K-nearest neighbors, SVM, Random Forest, and Naïve Bayes) for the classification of pneumonia using X-ray images. In order to improve the results, hyperparameter optimization was applied on chest X-ray dataset provided by Wang et al. [65].

Chouhan et al. [66] suggests an ensemble learning technique that connects outputs from all pertained deep learning architectures to identify the disease by applying the theory of transfer learning. The author utilized the dataset from the Guangzhou Women and Children's Medical Center [67], which includes a sum of 5232 images, 1346 of which associate with normal pneumonia. Out of the remaining 3883 images, 2538 image relate to bacterial and 1345 images relate to virus infections. The entire model hits a 96.4% output with a 99.62% recall from the dataset on secret information.

The performance of various CNN architectures on distinct abnormalities depending on the freely accessible OpenI dataset [68] observed that the same deep CNN model does not gives acceptable results for all type abnormalities [69]. However, ensemble learning substantially increased classification accuracy as compared to a single deep architecture. Eventually, the deep CNN models increased accuracy as compared to rule-based approaches.

Wang et al. [70] propose a deep learning model to retrieve visual features from CT Scan images for coronavirus classification. The study comprises of 1065 CT Scan images of patients, out of which 740 images are of viral pneumonia, and 325 images are of COVID-19. The design achieves an accuracy of 79.3%.

Xu et al. [71] present a 3-class design that can differentiate between COVID-19, viral, and normal instances. The segmentation-based procedure accomplishes an accuracy of 86.7% with 618 CT scan image samples (175 healthy, 224 Viral, and 110 COVID-19).

Gozes et al. [72] report a swift AI development cycle by means of a deep learning-based CT image analysis. Thus, the cited works in the literature use private datasets to develop a deep learning-based system for the diagnosis of COVID-19.

Apostolopoulos et al. [73] propose a three-class design that distinguishes among normal, viral, and COVID-19 instances using transfer learning techniques. The dataset comprises of 504 Normal, 700 Bacterial, and 224 COVID-19 X-ray images with sensitivity, specificity, and accuracy of 98.66%, 96.46%, and 96.78%, respectively. The data comprises of a small number of positive instances of COVID-19, so the results may vary significantly on a larger dataset.

In [74, 75], the authors present open-source datasets comprising of COVID-19 X-ray images. In [74], the scholars suggest a consolidated open-source dataset along with a deep CNN model known as COVID-Net for the classification of novel coronavirus. The dataset comprises of 8,066 normal, 5,538 pneumonia, and 358 coronavirus images. COVID-Net model applies CNN architecture with chest X-rays as inputs. The models attain an accuracy of 93.3% with limited COVID-19 images.

Afshar et al. [76] report a Capsule-based model, known as COVID-CAPS. The dataset contains 94,323 X-ray images of general thorax diseases. Extracted from the NIH repository, which consists of training and validation data with a split ratio of 0.9 : 0.1. The network achieves a specificity of 95.8%, accuracy of 95.7%, and sensitivity of 90%.

Abbas et al. [77] present a Convolution model that carries out dimensionality reduction to transform a high-dimensional feature space into a lower one. The dataset consists of 11 SARS, 80 Normal, and 105 coronavirus chest X-ray images. The design achieves an accuracy of 95.12%, specificity of 91.87%, and sensitivity of 97.91%.

Ucar and Korkmaz [78] fine-tune a pretrained model Squeeze Net using Bayesian optimization procedure to classify coronavirus-associated infections in X-ray images. The dataset comprises of 3895 Pneumonia, 66 COVID-19, and 1349 Normal chest X-ray images with a split ratio of 0.8 : 0.1 : 0.1. The design gives encouraging results on a minute dataset, which needs to be verified on a dataset with substantial number of COVID-19 images.

Khan et al. [79] apply the Xception pretrained deep model for automatically classifying COVID-19 images in chest X-rays. The dataset comprises of 284 coronaviruses, 327 viral, 330 bacterial pneumonia, and 310 normal images. The design achieves an accuracy of 87.5% and 95% for a 4-Class problem and 3-Class problem (COVID-19, Pneumonia, and Normal) on a small dataset.

Recent approaches also use lung segmentation [80], feature extraction using deep models, and texture descriptors [81] for the classification of COVID-19 in X-ray images. Another recent work [82] compares various techniques to deduce that patterns learned by neural networks do not correlate to the presence of coronavirus in X-ray images.

Most of the reported approaches depend on deep learning along with augmentation, fine-tuning, and ensemble learning for classification of pneumonia. These techniques have produced outstanding results and are now being used to identify novel coronavirus-associated infections in chest radiography. Most of the COVID-19 classification methods employed modest datasets to exhibit encouraging results, but there is no guarantee that these designs would generate similar outcomes on a more extensive dataset. Besides, many of the datasets employed in earlier studies are mostly unbalanced. In most of the approaches, cross-validation is not applied to check the generalization ability of the model. To our knowledge, ensemble learning is used by only a few studies (see Table 1).

In our approach, we have used a balanced and large dataset with 1050 images in each of the four classes. By applying approaches such as fine-tuning, transfer learning, augmentation, and ensemble learning in the said research, the proposed model is much more generalized and generates excellent results.

## 3. Materials and Methods

**Figure 2:**
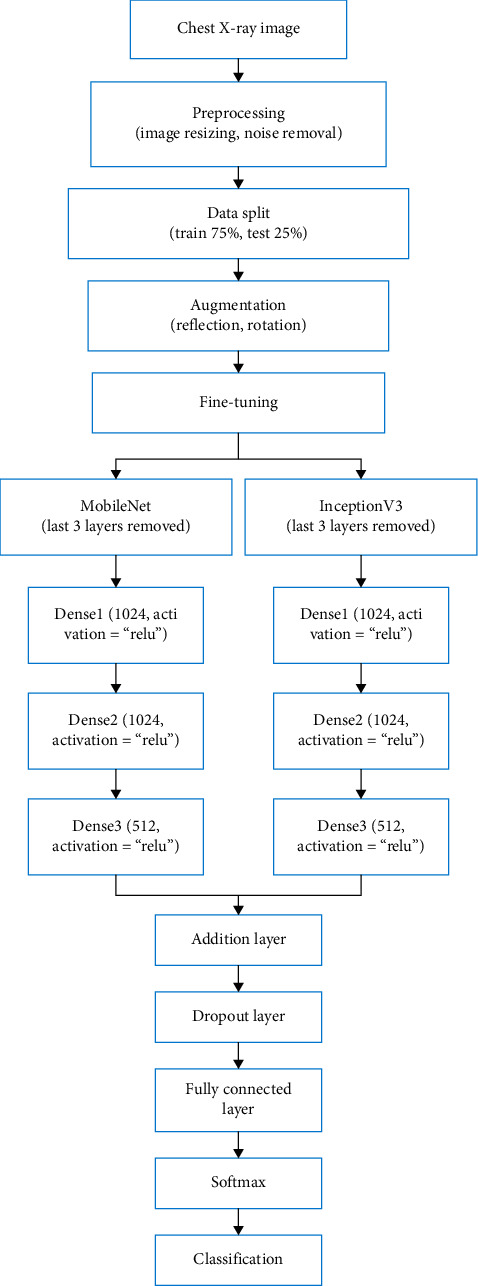
Various phases of novel coronavirus classification in chest X-ray images.

In the current study, transfer learning along with fine-tuning and augmentation are employed to pretrained deep models, to assess their performance. Then, a hybrid deep learning model using ensemble learning is proposed, which consists of MobileNet and InceptionV3 architectures. The ensemble learning model attains excellent performance on chest image dataset relating to chest-related infections (see Figure 2).

### 3.1. Data Description and Augmentation

The COVID dataset comprises of chest X-ray images pertaining to 4 classes (normal, bacterial, viral, and COVID-19). The dataset contains 4200 images, 1050 relating to each category. Images concerning three classes, i.e., (bacterial, viral, and normal) were retrieved from the Kaggle dataset [90]. Out of the total 1050 images of COVID-19, 912 were retrieved from an open-source Mendeley dataset [91], and the remaining were collected from three open-source repositories, namely, (i) Italian Society of Medical and Interventional Radiology (SIRM) [92], (ii) Radiopaedia [93], (iii) and github [75].

Augmentation is applied to the proposed model to enhance the volume of data, avoid overfitting [40, 68], and formulate a more generalized model. Several augmentation routines such as random rotation, random horizontal reflection, random vertical reflection, and range of horizontal and vertical shear are applied to generate an augmented dataset. The details of augmentation are as follows:

- Random rotation: the image rotates randomly in degrees according to the specified range
- Random horizontal reflection: the image is horizontally reflected from left to right with a probability of 50%
- Random vertical reflection: the image is vertically reflected from top to bottom with a probability of 50%
- Random horizontal shear: the image is horizontally shared in degrees according to the specified
- Random vertical shear: the image is vertically shared in degrees according to the specified

### 3.2. Preprocessing

Furthermore, the chest radiology images in the COVID-19 dataset are large in size, with some images with a dimension of 1007 × 1024 pixels. So, the images are adjusted according to the pretrained model utilized for classification. The mobile net and inceptionv3 have an input size of 224-by-224 and 299-by-299, respectively. The images are resized and supplied according to the chosen pretrained deep model. In some cases, the extended black background that surrounds an X-ray image is removed by cropping.

X-rays are mostly large-scale images, e.g., 1007 × 1024 pixels in grey scale. These images need to be resized and converted into 3-channel images. After this, they are acceptable as input to deep learning models. Various MATLAB built-in parameters and functions are used for performing preprocessing, the details of which are as follows:

- Resizing input images: input images of large-scale need to be adjusted according to the input scale of the deep CNN model. A function imresize () receives two parameters as input (image, new_image_dimension). As a result, the function returns a resized image.
- Converting a greyscale image into three-channel images: pretrained deep models use a colored image as input. In order to covert a grey-scale image into a three-channel image preprocess, _Xray () function is used, which receives an image as input and converts it into a three-channel image to adapt to the existing deep learning model.

### 3.3. Hyperparameter Tuning

In the current research, we tried to estimate the effect of numerous hyperparameters on the performance of the investigated pretrained models, such as the epochs, batch size, and learning rate. Specifically, we have evaluated three deep models: MobileNet, ResNet50, and InceptionV3. For each one of these deep models, we have performed fine-tuning over the subsequent parameters: learning rate = {3*e* − 6, 1*e* − 3, 1*e* − 4, 1*e* − 5, 4*e* − 6, 2*e* − 5, 1*e* − 8, 1*e* − 6, 2*e* − 5, 1*e* − 7}, batch size = {24, 32, 48, 64}, and epochs = {10, 20, 30, 40, 50, 60, 80, 100, 150, 160, 200, 300, 350}.

For deep models, numerous variations of hyperparameters were used, but mostly, these models exhibited good results with batch size of {32, 64}; however, for initial training epochs learning rate of {1*e* − 7, 1*e* − 6} was very productive, and slowly the learning rate was increased, and several variations were applied for various models. The details of different hyperparameters applied during training of various CNN models are shown in Table 2. To sustain a balanced utilization of the GPU resource capabilities, we have recognized the value 24 as the minimum batch size.

### 3.4. Overview of Convolutional Neural Networks (CNNs)

The background of CNN's depends on the theory of traditional Neural Networks. The convolution design consists of numerous building blocks, for example, convolution, pooling, and fully connected layers. A standard design comprises of replications of a pile of many convolution layers and a pooling layer, succeeded by one or more fully connected layers. Usually, after convolution operation, we generally perform pooling operation to decrease the dimensionality, which allows us to lessen the number of parameters that both reduce the training time and also contend overfitting. The pooling layer down sample's content feature maps, reducing the weight and height, along with keeping their salient features. The fully connected layers seek to attain midlevel features. Execution of a full connection in these layers requires a substantial number of weight parameters.

CNN's training commences in a feedforward manner, as it begins from the initial input layer to the final layer. Then, this error propagation begins in a reverse way as it starts from the final layer to the convolutional layer. Let **p** be the neural nodes in Layer **h** which accepts an input from the neural nodes **q** of layer **h** − 1 in the forward pass, calculated as given below:

(1) $$\begin{array}{c} ln_{p}^{h}=\sum_{q=1}^{n}W_{pq}^{h}x_{q}+b_{p}, \end{array}$$

where **b**_**p**_ and **W**_**pq**_^**h**^ are the bias term and weight vector of the **h**^th^ layer, respectively. The ReLU, a nonlinearity function, is used to compute the output as follows:

(2) $$\begin{array}{c} Out_{p}^{h}=Max\left(0,ln_{p}^{h}\right). \end{array}$$

All the neural nodes in convolution and fully connected layers use formulas (1) and (2) to compute the input and generate output in the shape of nonlinear activation. The pooling layer utilizes a *K* × *K* square window sliding on the *N* × *N* features map and takes the average or maximum value of the features inside the window. It, therefore, reduces the spatial dimension of the feature map from *N* × *N* to (*N* × *K*) × (*N* × *K*) as it generates a single value for *K* × *K* region.

The SoftMax function calculates the classification probability of every pathogen in the final layer, as given in equation (3), as follows:

(3) $$\begin{array}{c} Out_{p}^{h}=\frac{e^{ln_{p}^{h}}}{\sum_{k}e^{Out_{k}^{h}}}. \end{array}$$

A backpropagation procedure trains the CNN. This procedure minimizes the cost function for unknown weights *W*. The cost function is as follows:

(4) $$\begin{array}{c} C=-\frac{1}{m}\sum_{n=1}^{m}\text{ln}\left(p\left(y^{n}||X^{n}\right)\right). \end{array}$$

In equation (4), **m** represents the total number of training instances in a training set, *X*^*n*^ is the *n*^th^ instance in the training set and its label is *y*^*n*^, and the true classification probability is *p*(*y*^*n*^*|X*^*n*^).

### 3.5. Transfer Learning and Fine-Tuning Deep Learning Models

While training, the weights of layers of deep models are renewed after each iteration. There exist 314 layers and 25 million learnable parameters in the InceptionV3 design, whereas, in MobileNet, there exist 88 layers and 4.2 million parameters. For various pretrained architectures, there are a varied number of parameters and layers (see Table 3).

For the optimization and training of these CNN models, a substantial amount of data is vital. Though, for a relatively scantier dataset, it is quite challenging to learn the suitable local minimum for the cost function, as given in equation (4), and the model will experience overfitting. Thus, initially, weights are reclaimed from the InceptionV3 and MobileNet models. Following the weight transfer, we fine-tune MobileNet and InceptionV3 on the COVID-19 dataset by employing several variations of the batch size, learning rate, and the number of epochs. The primary layers in the pretrained models hold generic features, and the subsequent layers hold domain-specific features. In order to retain the features from primary layers intact and slow down learning in the remaining transferred layers, the initial learning rate is fixed to a minute value. Though, to learn faster in the recently added layers than in the transferred layers, the learning rate of the fully connected layer is set to a high value. The concluding fully connected layer of the architecture consists of 1000 neurons that harmonize to categories in the ImageNet dataset, so to acquire the domain-specific features of COVID-19 and various related infections in chest X-ray images, this layer is set to 4 neurons according to categories in the COVID-19_ dataset. The deep pretrained architectures, transfer learning, and ensemble learning are described in the subsequent sections.

#### 3.5.1. MobileNet

**Figure 3:**
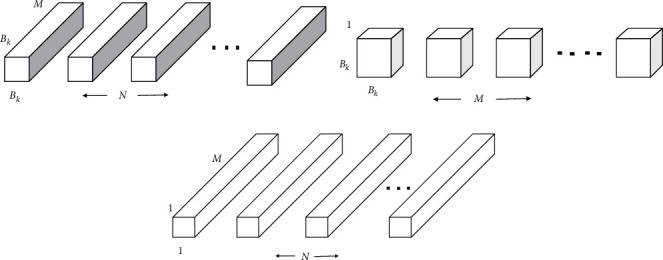
Representations of some convolution filters. (a) Standard convolution filters, (b) depthwise convolution filters, and (c) pointwise convolution filters.

MobileNet is a deep learning model for classification tasks, designed to maximize accuracy in devices with limited hardware resources. It is a small, low-power, and low-latency model, specified to encounter the resource limitations of a variety of use cases. It can perform classification, segmentation, detection, and embeddings like recently successful deep learning models, such as Inception [94]. In this part, we first elaborate on the main layers that MobileNet is dependent on, which are depthwise separable filters, and then explain its architecture:

*(1) Depthwise Separable Convolution*. The MobileNet architecture depends upon a depthwise separable convolution, which is a type of factorized convolution. It factorizes a regular convolution into a depthwise and 1 × 1 convolution filter known as pointwise convolution. For MobileNet, the depthwise convolution employs only one filter to every input channel. The pointwise convolution then employs a 1 × 1 convolution filter to blend the yields of the depthwise convolution. A standard convolution layer in one step performs filtering along with combining the input data into a new set of output data. The depthwise separable convolution separates this into two, one layer for filtering and another for merging. This step of factorization has the impact of substantially diminishing computing and design volume [94]. Diagram illustrates how a standard convolution layer is factorized among a depthwise convolution filter and a 1 × 1 convolution filter (see Figure 3).

*(2) Network Architecture*. The MobileNet model is assembled on depthwise separable convolution layers, as discussed in the preceding section apart from the 1^st^ layer, a fully connected convolution layer. By describing the model in such simplistic means, we can comfortably examine model topologies to discover a reliable model. The MobileNet architecture is shown in Table 4.

**Figure 4:**
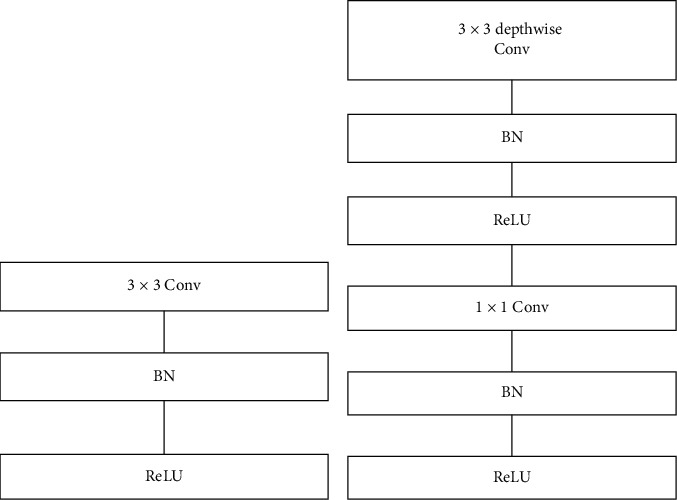
Difference between regular and depthwise convolutions: (a) regular convolution layer with batch-norm and ReLU and (b) depthwise separable convolution with depthwise and pointwise layers succeed by batch-norm and ReLU.

After each layer, there is a batch normalization, and ReLU, besides the last fully connected layer, which possesses no nonlinearity, is succeeded by a SoftMax layer for performing classification task. Diagram differs from layers with regular convolution, batch normalization, and ReLU to the factorized layers, with d-w convolution and 1 × 1 pointwise convolution, as well as batch-norm, and ReLU after every convolution layer is shown in Figure 4. Down sampling managed with stride convolution in the d-w convolution in addition to the initial layer. A concluding average pooling layer diminishes the spatial dimension to one before the fully connected layer. While considering pointwise and depthwise convolutions as distinct layers, the Mobile architecture processes 28-layers.

#### 3.5.2. InceptionV3

InceptionV3 is a convolutional model that consists of 48 layers. The model is trained on more than a million images from the ImageNet repository [43]. The pretrained model can classify images into 1000 classes. Consequently, the model learns rich features for a wide variety of images.

**Figure 5:**
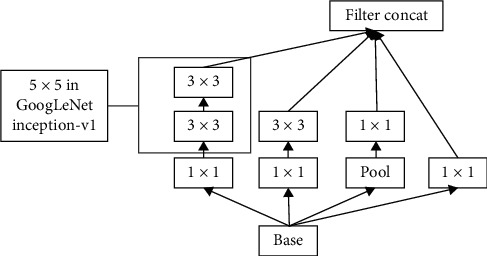
InceptionV3 module.

InceptionV3 is an improved version of inceptionV2 that accomplishes immense proficiency in performing image classification tasks by factorizing 5 × 5 convolution layer into two more simple 3 × 3 convolution layers. The representational bottleneck is removed by appending a regularization portion to the loss procedure. The unique InceptionV3 design limits overfitting and accomplishes label smoothing to a great extent. The architecture also factorizes a 7 × 7 convolution layer and joins several distinct deep CNN layers with batch normalization technique, producing even greater accuracy with less computational complexity. Diagram demonstrates the detailed structure of the InceptionV3 module (see Figure 5).

#### 3.5.3. Ensemble Classification

These types of CNNs are nonlinear designs that learn complicated associations from the input data with the help of stochastic optimization and backpropagation which makes them extremely susceptible to arbitrary weight initializations and the noise existing in the training dataset. These concerns can be mitigated by applying ensemble approach by training various deep learning architectures and merging their predictions, where a specific model's deficiencies are balanced by the forecasts of the additional model. Joined predictions are proven to be better than individual deep architectures [89]. There exist numerous ensembles learning approaches stated in the research studies, including simple and blending, stacking, max voting, boosting, weighted averaging, and various others that decrease the variance error and enhance performance and generalization ability of deep pretrained models. When implemented to chest X-rays, the contributors of [58, 95, 96] leveraged the usage of an ensemble learning for tuberculosis classification in X-rays can help in attaining better results. An averaging ensemble learning technique applied to pretrained deep models helped researchers of [69] toward enhancing cardiomegaly classification utilizing chest X-rays.

Initially, we perform feature extraction from the two best-performing models, i.e., (MobileNet and InceptionV3). However, before extracting features, three dense layers are added to these selected models, which help the model in learning complex features. The subsequent addition layers help in feature fusion from these deep models. Then, we add a 0.5 dropout layer, before classification, which helps in addressing the challenges of long training time and overfitting [97].

### 3.6. Experiments

#### 3.6.1. Software and Hardware

The current approach applied several pretrained such as MobileNet and InceptionV3 using a MATLAB R2019b and NIVIDA GeForce-2070 GPU with 8 GB of DDR6 onboard memory with 14 Gbps of frequency, and 2304 cores with a frequency of 1620 MHz's. The system also consists of 16 GB DDR4, and a 1 TB SSD hard drive that further improves system speed. These pretrained deep models are available online and can be installed/downloaded from the MATLAB website using the Add-On Explorer.

#### 3.6.2. Performance Measures

To compare the different deep models with the proposed methodology, we use various performance measures such as precision, recall, and FScore along with accuracy, as accuracy alone cannot determine the effectiveness of a model [98]. During the experimentation, 75% of the dataset is for training the model, and 25% is for testing purposes. A 4-fold cross-validation is applied to verify the performance of the proposed methodology. These cross-validation results are averaged to produce performance measures such as accuracy and FScore.

The accuracy of a model calculates how correctly the scores are forecasted. The precision learns the reproducibility of the measure or the correct predictions. Recall determines the correct results. *F*-score utilizes precision and recall to calculate an averaging of both scores. The following equations display how to compute these metrics, where TP, TN, FP, and FN are true-positive, true-negative, false-positive, and false-negative, respectively:

(5) $$\begin{array}{c} \text{Accuracy}=\frac{\text{TN}+\text{TP}}{\text{FP}+\text{TP}+\text{FN}+\text{TN}},\\ \text{Precision}=\frac{\text{TP}}{\text{FP}+\text{TP}},\\ \text{Recall}=\frac{\text{TP}}{\text{FN}+\text{TP}},\\ \text{FScore}=\frac{2\times \text{Precision}\times \text{Recall}}{\text{Precision}+\text{Recall}}. \end{array}$$

#### 3.6.3. Experimental Strategies

Various experimental strategies are adopted to show the effect of fine-tuning and augmentation on deep learning models with pretrained weight from ImageNet and without these prelearned weights. These strategies are as follows:

- Deep models trained from scratch on the target dataset without any previously learned weights from ImageNet dataset
- Fine-tuning deep models on the original target dataset without applying any augmentation strategies while keeping pretrained weights and all the layers of the model unfrozen
- Fine-tuning deep models on target dataset while applying augmentation strategies, along with keeping pretrained weights and all the layers of the model unfrozen

## 4. Results and Discussion

The section exhibits the experimental results of CNN models along with discussing the improvement these approaches, i.e., ensemble earning, augmentation, and transfer learning , have brought in the proposed methodology. Initially, we choose the two best-accomplishing deep models and extract features from these models by applying the ensemble learning approach. Then, for classification, a fully connected layer, SoftMax layer, and a classification layer are added to the hybrid ensemble model. Firstly, we will examine the performance of these deep learning models in three different aspects when these models are (i) trained from the beginning, (ii) fine-tuned with all layers unfrozen, and (iii) augmentation and fine-tuned with all layers unfrozen. For each approach, precision, recall, FScore, and accuracy are calculated for deep architectures on the COVID-19 dataset (see Table 5).

**Figure 6:**
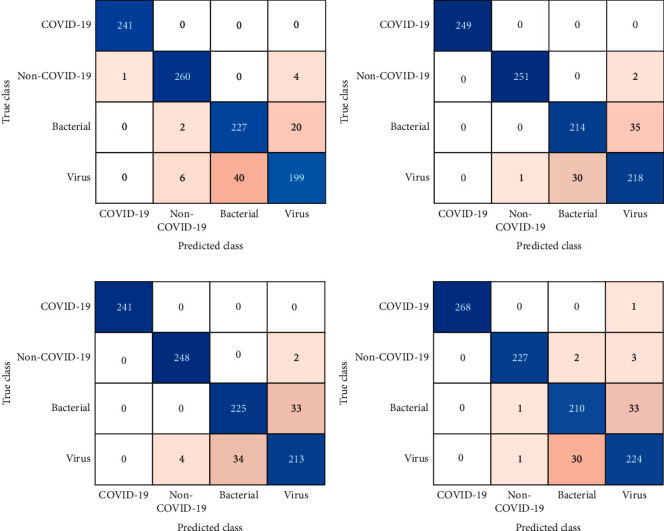
Confusion matrices of ensemble learning model for test dataset of 4-folds cross-validation: (a) fold-1, (b) fold-2, (c) fold-3, and (d) fold-4.

**Figure 7:**
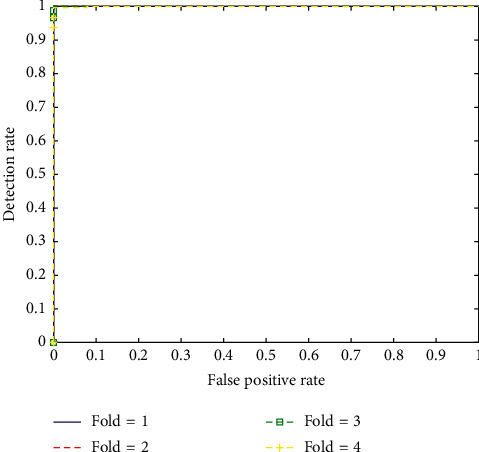
ROC curve for classification by deep ensemble model for 4-folds of test dataset.

By analyzing these matrices of deep models on the COVID-19 dataset, we can conclude the following results:

1. In respect to this small dataset, popular shallow classification models generate significantly better than deeper models, as the classification matrices of these deep models trained from scratch depict. These deep models generate relatively low precision, recall, FScore, and accuracy because they have not been fully trained due to an enormous number of parameters and insufficient training data. Previous works also report that shallow models display better results than deeper models for image classification tasks [55].
2. Fine-tuning is utilized on multiple pretrained CNN models using Chest X-ray images, to help deep models converge swiftly and acquire features related to a specific domain. It can also help improve the accuracy and FScore of these deep models, even if trained from scratch during image classification tasks. As the results portray that the models only fine-tuned on the original COVID-19 dataset can substantially enhance accuracy and FScore, even if the model is trained from scratch. Previous studies also reveal that fine-tuning a deep model is essential for its reusability [99]. Recent research studies have confirmed that fine-tuning is effective for different types of classification issues in the biological field [100].
3. Transfer learning is applied in all our approaches except training the model from scratch, which shows that wherever transfer learning is applied, there is a substantial increase in the performance of all matrices. Some notable investigations [92, 93, 101] by researchers augment our viewpoint that transfer learning can generate outstanding outcomes, especially in the case of small datasets [55, 92].
4. Augmentation is also quite useful for increasing a model's performance, especially when the dataset is small. The convolution models, along with conventional augmentation procedures, can make pretrained CNN models achieve enhanced performance. As exhibited in the results, in the approach where augmentation is employed, all CNN models observed almost 1–3% increase in precision, 1–2.5% increase in recall, 1–2.5% FScore, and 0.5–1.1% increase in accuracy over former fine-tuned CNN models without augmentation. Investigations also maintain our opinion that augmentation can help in increasing the performance and producing a more generalized prototype without the menace of overfitting [55, 102].
5. In the proposed ensemble model, two prime deep models (MobileNet and InceptionV3) selected for ensemble learning produced accuracy and FScore of 95.18% and 90.34% and 91.47% and 95.75%, respectively. MobileNet depends on a streamlined architectural design that applies depthwise separable convolutions with different layers for filtering and merging. The factorization has the impact of substantially diminishing computational cost and design dimensions [94]. Such type of network possesses lesser number of parameters to adjust, as compared to standard convolution networks, which reduces overfitting. A recent investigation manifested that the InceptionV3 model, fine-tuned using chest X-ray films relating to the examination of pulmonary nodules, accomplished fantastic results for the diagnosis of thoracic disease, similar to the conclusion of expert radiologists [103]. Another research also applies transfer learning and deep model such as InceptionV3 on chest X-rays for the classification of pneumonia [66].Its architecture utilizes factorized inception blocks, facilitating the interface to pick appropriate kernel sizes for the convolution layers, which allows the design to gain both high- and low-level features with larger and smaller convolution layers [104].
6. The proposed ensemble model generates precision, recall, FScore, and accuracy of 93.01, 92.97, 92.97, and 96.49%, respectively, while applying fine-tuning and augmentation strategies. The model produces a better precision, recall, FScore, and accuracy as compared to any of the individual deep models. The proposed model attained almost 1–4.2% increase in precision, 2–5% increase in recall, 2–5% increase in FScore, and 1–2.2% increase in accuracy over all the employed CNN models (see Table 5). Various research studies propose that contrary to the conventional CNN models, ensemble learning models by merging deep CNNs acquire more useful features from images in the training data. These ensemble models have accomplished outstanding results in image classification tasks in various domains [95, 105], along with pneumonia classification [66], cardiovascular tissues identification [106], and especially in the area of radiology images [89, 97].
7. For the test dataset, which was kept separately, the model produced precision, recall, FScore, and accuracy of 89.93%, 88.38%, 88.64%, and 94.19%, respectively.
8. The confusion matrices further elaborates the test results of the proposed model for COVID-19 dataset (see Figure 6). The first matrix of Fold-1 shows that there are five misclassifications in Normal and no misclassification in the COVID-19 class. However, misclassification is relatively high in the other two classes (bacterial 22 and viral 46). Similarly, in all the four-folds, there is only one misclassification in the COVID-19 class and fourteen misclassifications in the Normal class.
9. Plot shows ROC curves for all the four-folds of the COVID-19 dataset. These curves are plotted to further analyze the performance of the presented methodology on test dataset (see Figure 7). More than 50% area under the curve gives acceptable performance and area about 100% represents best performance. The ROC curves in Figure 7 reflect that the model generated excellent results for all the four-folds of COVID-19 dataset.

Overfitting can be a significant challenge, especially with inadequate training examples. The design might accomplish substantial training accuracy, but when tested for unseen real-world data, it may not generalize well for new instances. So, a significant issue to investigate is that whether any overfitting or the proposed model has generalized well for supplied instances. To perform this comparison, we estimate the performance of the ensemble model by assessing the gap between the validation and training curves amongst the no of epochs. Wider the space among the curve, the higher the overfitting.

**Figure 8:**
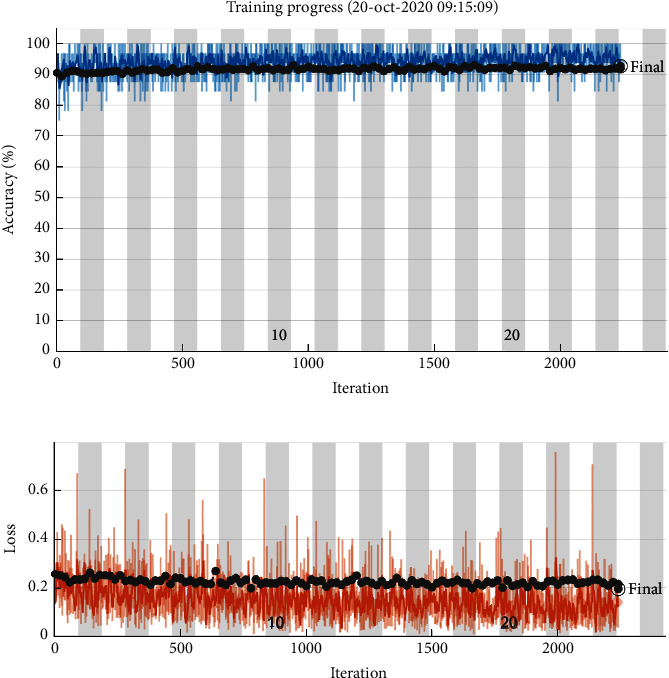
Learning curves for (a) training and validation accuracy (blue, black doted lines) and (b) training and validation loss (orange, black doted lines) of fold-3 of fine-tuned pretrained ensemble model, for novel coronavirus classification using chest X-rays.

Plot displays the variation in accuracy and loss among training and validation curves of the deep model, as the number of epochs is varied (see Figure 8). The training curve relates to fold-3 of the model, which was further trained to increase the accuracy. After training for 25 epochs, there was a slight increase in the accuracy and decrease in loss. The figure also illustrates that the validation and train curves proceed side by side without a gap, which indicates that there is no overfitting, and the ensemble model has generalized properly over the provided instances.

## 5. Conclusion

In the recent study, a deep coronavirus classification technique is presented, which takes the benefit of ensemble learning, fine-tuning, data augmentation, and transfer learning to distinguish among four different categories of chest-related infections using a larger and balanced dataset. Ensemble learning helps in merging the qualities of different models while overcoming deficiencies of individual models. Fine-tuning facilitates the model converge swiftly and acquire domain-specific features. Data augmentation makes training datasets more versatile, which improves the generalization capability of the design and thus helps in managing the overfitting issue. Transfer learning addresses the need for a substantial amount of training data. The proposed deep learning design consists of MobileNet and InceptionV3 architectures, which generates far better classification results than any of the selected fine-tuned pretrained models. The final proposed model achieved precision, recall, FScore, and accuracy of 93.01%, 92.97%, 92.97%, and 96.49%, respectively. For test dataset, the model attained precision, recall, FScore, and accuracy of 89.93%, 88.38%, 88.64%, and 94.19%, respectively, which can significantly help radiologists and diagnostic staff in the correct identification of the novel coronavirus pathogen in chest X-rays. The swift and computer-aided diagnosis using our model can help in saving precious human lives and thereby decreasing the socio-economic effect on civic society.

However, the dataset is still not sufficient for a highly accurate and practical deep learning solution that could be acceptable as a benchmark for identifying COVID-19 infections in patients from X-ray images. In future work, as an effort to further improve the classification accuracy, FScore of the model will be carried out, while utilizing significantly deeper models trained and tested on substantially larger dataset. Subsequently, the increase in classification accuracy and FScore will improve the reliability and efficiency of the model.

## Figures and Tables

**Table 1:** Comparison of numerous approaches applied for COVID-19 classification.

Approach	Classes	Dataset details (normal, N; bacterial, B; viral, V; tuberculosis, T; pneumonia, P; unknown, UP; COVID-19, C)	Data augmentation	Transfer learning	Balanced dataset	Cross-validation	Ensemble learning
Oh et al. [83]	5	N 191, B 54, T 57, V 20, C 180	✓	✓	×	×	×
Wang et al. [74]	3	N 8066, P 5538, C 358	✓	×	×	×	×
Minaee et al. [84]	2	N 5000, C 184	✓	×	×	×	×
Afshar et al. [76]	5	Not specified	×	✓	×	×	×
Luz et al. [85]	3	N 8066, P 5521, C 183	✓	✓	×	×	×
Khobahi et al. [86]	3	N 8851, P 9579, C 99	✓	✓	×	×	×
Ucar and Korkmaz [78]	3	N 1349, P 3895, C 66	✓	✓	×	×	×
Farooq and Hafiz [87]	4	N 1203, B 931, V 660, C 68	✓	✓	×	×	×
Chowdhury et al. [88]	3	N 1579, V 1485, C 423	✓	✓	×	✓	×
Rajaraman et al. [89]	4	N 7595, B 2780, C 313, UP 6012	×	✓	×	×	✓

**Table 2:** The detail of hyperparameters for different pretrained models.

Deep models	Batch size	Learning rate
MobileNet	48, 32, 24, 64	1*e* − 6, 1*e* − 8, 2*e* − 5, 4*e* − 6, 1*e* − 7, 1*e* − 5, 3*e* − 6
InceptionV3	32, 48, 24, 64	1*e* − 3, 1*e* − 6, 1*e* − 7, 4*e* − 6
InceptioV3-MobileNet	24, 32, 48, 64	1*e* − 8, 1*e* − 3, 1*e* − 7, 4*e* − 6, 1*e* − 6, 1*e* − 4, 1*e* − 5

**Table 3:** The detail of layers and parameters for different pretrained models.

Pretrained model	Layers	Parameters (weights) (million)
MobileNet	88	4.2
ResNet50	50	25.6
inceptionV3	316	25

**Table 4:** MobileNet detail architecture.

Convolution type/strides	Size of filter	Shape of input
Con/s-2	(3 × 3 × 3 × 32)	(224 × 224 × 3)
Con d-w/s-1	(3 × 3 × 32) d-w	(112 × 112 × 32)
Con/s-1	(1 × 1 × 32 × 64)	(112 × 112 × 32)
Con d-w/s-2	(3 × 3 × 64) d-w	(112 × 112 × 64)
Con/s-1	(1 × 1 × 64 × 128)	(56 × 56 × 64)
Con d-w/s-1	(3 × 3 × 128) d-w	(56 × 56 × 128)
Con/s-1	(1 × 1 × 128 × 128)	(56 × 56 × 128)
Con d-w/s-2	(3 × 3 × 128) d-w	(56 × 56 × 128)
Con/s-1	(1 × 1 × 128 × 256)	(28 × 28 × 128)
Con d-w/s-1	(3 × 3 × 256) d-w	(28 × 28 × 256)
Con/s-1	(1 × 1 × 256 × 256)	(28 × 28 × 256)
Con d-w/s-2	(3 × 3 × 256) d-w	(28 × 28 × 256)
Con/s-1	(1 × 1 × 256 × 512)	(14 × 14 × 256)
5 × Conv d-w/s-1 Conv/s-1	(3 × 3 × 512) d-w (1 × 1 × 512 × 512)	(14 × 14 × 512) (14 × 14 × 512)
Con d-w/s-2	(3 × 3 × 512) d-w	(14 × 14 × 512)
Con/s-1	(1 × 1 × 512 × 1024)	(7 × 7 × 512)
Con d-w/s-2	(3 × 3 × 1024) d-w	(7 × 7 × 1024)
Con/s-1	(1 × 1 × 1024 × 1024)	(7 × 7 × 1024)
Avg pool/s-1	Pool (7 × 7)	(7 × 7 × 1024)
FC/s-1	(1024 × 1000)	(1 × 1 × 1024)
SoftMax/s-1	Classifier	(1 × 1 × 1000)

**Table 5:** A comparison of deep ensemble model with several techniques applied on deep learning models.

Model types	Methods	Precision	Recall	FScore	Accuracy
*InceptionV3_MobileNet Model*		93.01 ± 0.24	92.97 ± 0.28	92.97 ± 0.29	96.49 ± 0.11

*MobileNet*	FT on ODS-TFB	67.13 ± 4.16	66.66 ± 3.86	66.62 ± 3.90	83.34 ± 2.10
	FT on ODS-ALUF	89.49 ± 2.08	89.22 ± 2.43	89.16 ± 2.51	94.60 ± 1.21
	FT on ADS-ALUF	90.44 ± 0.69	90.35 ± 0.76	90.34 ± 0.75	95.18 ± 0.39

*ResNet50*	FT on ODS-TFB	60.09 ± 11.27	56.87 ± 13.86	56.28 ± 14.81	78.40 ± 6.84
	FT on ODS-ALUF	86.95 ± 1.51	86.67 ± 1.91	86.55 ± 1.81	93.33 ± 0.92
	FT on ADS-ALUF	89.27 ± 1.96	88.73 ± 2.15	88.73 ± 2.16	94.39 ± 1.03

*InceptionV3*	FT on ODS-TFB	59.41 ± 12.43	56.92 ± 13.17	57.38 ± 12.99	78.49 ± 6.73
	FT on ODS-ALUF	91.13 ± 0.94	90.94 ± 0.90	90.95 ± 0.84	95.48 ± 0.47
	FT on ADS-ALUF	91.98 ± 0.78	91.51 ± 1.35	91.47 ± 1.38	95.75 ± 0.68

ODS, ADS, TFB, ALUF, and FT stand for original dataset, augmented dataset, training from beginning, all layers unfrozen, and fine-tuned.

## Data Availability

The data used to support the findings of the study are available from the corresponding author upon request.
