# Supplementary material for: Deep Ensemble Model for Classification of Novel Coronavirus in Chest X-Ray Images
Source: Comput Intell Neurosci. 2021 Jan 5;2021:8890226. doi: 10.1155/2021/8890226 (PMC7805527; doi:10.1155/2021/8890226)
Supplement: Supplementary Materials — COVID-19 dataset is provided as supplementary material, consisting of chest X-ray images. [file 8890226.f1.docx]

8890226: Supplementary Materials

Link for dataset

<https://drive.google.com/file/d/1OvTIgGecfhai8Ri0jHy-muG6XcnHcJuZ/view?usp=sharing>

trained Model

<https://drive.google.com/file/d/1-7WJg7srlsgRsycuQQ9EmaqUKVvKD_hf/view?usp=sharing>
